# *In vitro* effects of different biomaterials on canine dental pulp stem cells

**DOI:** 10.3389/fvets.2026.1758525

**Published:** 2026-02-05

**Authors:** Robert Marx, Ana Nemec, Andraž Kocjan, Metka Voga

**Affiliations:** 1 Animal Hospital Hofheim, IVC Evidensia, Hofheim, Germany; 2 Small Animal Clinic, Veterinary Faculty, University of Ljubljana, Ljubljana, Slovenia; 3 Department for Nanostructured Materials, Jožef Stefan Institute, Ljubljana, Slovenia; 4 Clinic for Reproduction and Large Animals, Veterinary Faculty, University of Ljubljana, Ljubljana, Slovenia

**Keywords:** acute cytotoxicity, calciumsilicate-based biomaterials, canine dental pulp stem cells, cell-material applications, veterinary regenerative endodontics, MTA, RS, CellFoam

## Abstract

**Objective:**

Regenerative endodontic treatments are being developed in veterinary dentistry. The aim of this study was to evaluate the biocompatibility and odontogenic potential of three biomaterials, ProRoot^®^ MTA (MTA), RS + ™ (RS+), and CellFoam™ (CF), on canine dental pulp stem cells (cDPSCs) under conditions simulating early and clinically relevant exposures.

**Methods:**

cDPSCs were isolated from three healthy dog teeth extracted for clinical reasons and characterized by flow cytometry (CD44^+^/CD90^+^/CD29^+^/CD34^−^) and multilineage differentiation. Cells were cultured with material suspensions (acute cytotoxic effect) or conditioned medium (physiologically relevant effect). Metabolic activity and cell viability were assessed by MTT and live/dead assays. Osteogenic/odontogenic differentiation was evaluated by Alizarin Red S staining and RT–qPCR for RUNX2, ALPL, and MMP13 expression.

**Results:**

In suspension cultures, compared with MTA and RS+, CF maintained significantly higher metabolic activity and cell viability across several dilutions, indicating lower acute cytotoxicity. Under conditioned exposure, no significant differences among materials were observed, reflecting the dilution and buffering effects that mitigate early reactivity. All the materials supported Alizarin Red S-positive mineral deposition, with a significant difference at D3, when ARS staining of cDPSCs was greater in cells conditioned with MTA than in those conditioned with CF. Gene expression analysis revealed lower RUNX2 and ALPL expression in MTA-conditioned cells, suggesting, together with ARS staining, progression toward a more advanced osteogenic or odontogenic differentiation stage. MMP13 expression remained comparable across materials.

**Conclusion:**

MTA, RS+, and CF demonstrated overall biocompatibility with cDPSCs and supported odontogenic differentiation under clinically relevant conditions. CF exhibited the lowest acute cytotoxicity, indicating its potential as a carrier for DPSC-based regenerative endodontic applications. These findings support the translational importance of *in vitro* cDPSC models for evaluating biomaterial performance in veterinary regenerative endodontics.

## 1 Introduction

Traumatic dentoalveolar injuries (TDIs) are common in veterinary dental practice, affecting an estimated 26.2% of patients (92.7% in dogs and 7.3% in cats), with complicated crown fractures (CCFs) representing the most frequent entity (1). Following pulp exposure, superficial inflammation is evident histologically within 48 h; infection typically spreads throughout the pulp, leading to necrosis by approximately 65 days, and apical periodontitis can be observed in dogs within 20 days of exposure (2, 3).

Endodontically compromised teeth require intervention to eradicate infection and alleviate pain. Management options include extraction or endodontic therapy aimed at eliminating the intraradicular microbial ecosystem (4–8). When endodontic treatment is selected for a vital tooth, amputation of approximately 5 mm of the coronal-most dental pulp within (ideally) the first 48 h in mature permanent teeth suffices to remove infected and inflamed pulp, followed by appropriate medication placement and restoration (i.e., vital pulpectomy, VP). Vital pulpectomy is recommended whenever feasible for immature permanent teeth with open apices (8–12). When the tooth becomes irreversibly inflamed or nonvital, root canal treatment (RCT) remains an endodontic treatment option for mature teeth. Endodontic treatment of nonvital immature permanent teeth remains challenging, but regenerative endodontic treatments are also being developed in veterinary dentistry (13, 14).

The success of such treatments depends on materials that are not only bioactive and capable of forming a mineralized barrier but also biocompatible with dental pulp stem cells (DPSCs) and supportive of their differentiation potential (15, 16). Historically, calcium hydroxide was used as a pulp dressing for VP; however, mineral trioxide aggregate (MTA) has demonstrated superior outcomes in dogs and is now widely considered the standard material in this context (12, 17, 18). Nevertheless, classic MTA is a Portland cement-based endodontic material containing several oxides and radiopaque, brownish-colored bismuth oxide (Bi_2_O_3_), associated with a relatively long setting time (3–4 h), higher cost, and handling challenges; newer, bioceramic MTA-like hydraulic calcium trisilicate cements have been developed via purer, synthetic routes to mitigate these drawbacks, where Bi_2_O_3_ is usually replaced with biocompatible zirconia (19–22). Across these modalities, the ability of materials to promote a durable protective barrier while preserving vital pulp or enabling pulp regeneration is central to clinical success (17, 23), underscoring the need to evaluate the biocompatibility and bioactivity toward the cellular components of dental pulp (17, 24, 25).

Recent advances in regenerative dental medicine have introduced the concept of combining bioactive materials with stem cell-based approaches to achieve true pulp regeneration rather than mere repair (26–28). DPSCs represent a promising cell source and are particularly relevant for modeling vital pulp therapy and regenerative endodontic procedures in veterinary patients, as they are resident within dental soft tissues and contribute to dentin–pulp complex repair and regeneration (29). Using DPSCs, we can evaluate how biomaterials interact with resident DPSCs under clinically relevant *in vitro* conditions while also assessing how different materials influence stem cell behavior for potential combined cell–material applications. Emerging therapies that combine the reparative potential of DPSCs with biocompatible scaffolds or cements offer promise for treating pulp injuries (15).

The aim of our study was to investigate the *in vitro* effects of three biomaterials—ProRoot^®^ MTA (MTA), RS + ™ (RS+) and CellFoam™ (CF)—on cDPSCs to clarify the influence of these materials on cDPSCs from two different but complementary aspects:

1. Acute cytotoxic effect—the initial acute effect of particle-associated cytotoxicity that may occur immediately after material placement, simulating the initial contact between freshly mixed material and the surrounding pulp cells.
2. Physiologically relevant effect—a longer-term effect of soluble leachates, simulating more physiological, diffusion-controlled conditions, representing the environment that cells experience within the tissue or when the material is combined with stem cell-laden scaffolds in regenerative applications.

In this study, we investigated how selected biomaterials (with MTA as the clinical benchmark reference material in veterinary endodontics) affect cDPSCs with respect to viability, metabolic activity, and odontogenic/osteogenic differentiation potential *in vitro* to inform their prospective therapeutic use in veterinary endodontics.

We hypothesized that the biomaterials RS + ™ and CellFoam™ exhibit *in vitro* biocompatibility comparable to that of ProRoot^®^ MTA when applied to canine dental pulp stem cells with respect to cell viability, metabolic activity, and odontogenic/osteogenic differentiation potential.

## 2 Materials and methods

### 2.1 Dental pulp tissue collection

Dental pulp tissue was collected at the Small Animal Clinic, Veterinary Faculty, University of Ljubljana, from the teeth of two client-owned dogs undergoing clinically indicated mandibular canine tooth extraction of endodontally and periodontally healthy teeth (to treat traumatic malocclusion from linguoversion) under general anesthesia. Dogs (a 4-year-old male Poodle and a 7-month-old male Labrador Retriever) were treated by a board-certified veterinary dentist in accordance with the current state-of-the-art guidelines; no changes to the treatment protocols were made for the purpose of the study. Owners provided written informed consent for the procedures. Immediately after surgical extraction (30), all three teeth were disinfected externally (i.e., briefly rinsed in 2% chlorhexidine), the crowns were sectioned under aseptic conditions to expose the pulp chamber, and pulp tissue was retrieved with a sterile barbed broach and transferred into cold Dulbecco’s phosphate-buffered saline (DPBS; Gibco, Grand Island, NY, USA).

### 2.2 Isolation and expansion of canine dental pulp stem cells (cDPSCs)

The three isolated dental pulp tissue samples were subsequently washed with DPBS (Gibco, Grand Island, NY, USA), cut into small pieces with a scalpel and incubated overnight at 37 °C in Dulbecco’s modified Eagle’s medium (DMEM, Gibco, Grand Island, NY, USA) supplemented with 0.1% collagenase type II (Sigma–Aldrich, Taufkirchen, Germany). The digested tissue was centrifuged at 240 × *g* for 4 min, after which the supernatant was discarded. The cell pellet was resuspended in cell culture medium supplemented with DMEM, 10% fetal bovine serum (FBS; Gibco, Grand Island, NY, USA) and 1% antibiotic (Penicillin: Streptomycin solution 100X, VWR International, Vienna, Austria). The cell suspension was plated into 6-well plates (TPP, Trasadingen, Switzerland) at passage 0 and cultured at 37 °C in a 5% CO_2_ incubator. The cell culture medium was changed every 2–3 days. After reaching 70–90% confluence, the cells were trypsinized and multiplied by seeding into a larger (T75) cell culture flask at passage 1. After a sufficient number of cells were obtained, the cells from passage 1 were frozen at −80 °C in cell freezing medium containing 10% dimethylsulfoxide (Sigma–Aldrich, Taufkirchen, Germany). Thawed cells were seeded at passage 2, multiplied and further processed for the expression of surface markers, differentiation potential, and experimental cell cultures (Table 1). Suspension media cultures were used for MTT and live/dead assays, and conditioned media cultures were used for MTT and live/dead assays, Alizarin Red S staining and gene expression analysis.

**Table 1:** Experimental groups, media, and culture conditions used in the study (applies to MTA, RS+, and CF media conditions).

Experimental medium (24 h-cell pretreatment)	Control of the culture	Dilutions (D) of experimental medium	Analysis (Method)
Suspension MTA/RS+/CF medium	Negative control (nontreated cells)	D1–D8	Viability (live/dead assay) Metabolic activity (MTT)
Conditioned MTA/RS+/CF medium	Negative control (non-treated cells) for non-differentiated cells and Positive control (non-treated differentiated cells) for differentiated cells	D1–D4	Viability (live/dead assay) Metabolic activity (MTT) Mineralization (Alizarin Red S) Gene expression analysis (qPCR)

D1 = 5%, followed by twofold serial dilutions.

### 2.3 Flow cytometry for cell-surface markers

Flow cytometry was performed on the untreated cells to evaluate the expression of cell surface markers. Antibodies against the MSC markers CD44, CD90, CD29, and CD34 were applied as previously reported for cDPSCs (CD44^+^/CD90^+^/CD29^+^/CD34^−^) (31, 32). A total of 1 × 10^6^ cells were used. Cells frozen at passage 1 were thawed, seeded at passage 2, multiplied, reseeded and analyzed at passage 3. Following trypsinization, the cells were counted, centrifuged (240 × *g* for 4 min), and washed twice with DPBS. Cells were stained with the following antibodies for canine adipose-derived mesenchymal stem cells (ADMSCs): allophycocyanin (APC) conjugated against CD44 (antibody clone IM7, 103,012, BioLegend, San Diego, CA, USA), phycoerythrin (PE) conjugated against CD90 (antibody clone YKIX337.217, 12–5,900-42, eBioscience, San Diego, CA, USA), fluorescein isothiocyanate (FITC) conjugated against CD29 (antibody clone MEM-101A, MA1-19566, Thermo Fisher Scientific, Waltham, MA, USA), and CD34 (antibody clone 581, 60013FI, Stemcell Technologies, Cambridge, MA, USA). For antibody titration, 1, 2, 3, 4, 5, and 10 μL of each antiserum per 100 μL of 1 × 10^6^ cells was used. Appropriate dilutions of the antibodies used for staining are shown in Table 2. The cells were then vortexed, incubated at room temperature in the dark for 10 min, washed twice with DPBS, vortexed, and centrifuged again (240 × *g* for 5 min). The supernatant was subsequently decanted. Finally, the cells were resuspended in 100 μL of DPBS for FACS analysis. The exclusion of nonviable cells was performed by staining cells with propidium iodide solution (Molecular Probes, Eugene, OR, USA). Experimental settings were set up using unstained cells and single-color staining. A minimum of 20,000 events was recorded. The cells were analyzed with a BD FACSAria III flow cytometer (BD Bioscience, Franklin Lakes, NJ, USA). FACSDiva 9.4 software (BD Bioscience) was used for FACS data analysis.

**Table 2:** Antibodies and dilutions used for flow cytometry.

Surface marker	Conjugation	Antibody clone	Isotype	Target species	Catalog no.	Source	Antibody dilution per 1 × 10^6^ cells
CD44	APC	IM7	Rat IgG2b	Mouse, Human	103,012	BioLegend (USA)	1:67
CD90	PE	YKIX337.217	Mouse IgG1	Dog	12–5,900-42	eBioscience (USA)	1:20
CD29	FITC	MEM-101A	Mouse IgG1	Dog/Human/Pig	MA1-19566	ThermoFisher Scientific (USA)	1:5
CD34	FITC	581	Mouse IgG1	Human	60013FI	STEMCELL Technologies (Canada)	1:20

CD, cluster of differentiation; FITC, fluorescein isothiocyanate; APC, allophycocyanin; PE, phycoerythrin.

### 2.4 Differentiation potential

For the determination of differentiation potential, untreated cells were used. Differentiation potential was assessed by inducing cell differentiation into osteocytes and chondrocytes. Cells frozen at passage 1 were thawed, seeded at passage 2, multiplied and reseeded at passage 3 for the differentiation assay. For osteogenic differentiation, 4 × 10^4^ cells were seeded in 12-well plates. After 90–100% confluence was reached, the cell culture medium was removed. Osteogenic medium (StemPro Osteogenesis Differentiation Kit, Gibco, Grand Island, NY, USA) was added, and the medium was changed every 2–3 days. The cell culture medium was added to the wells, which served as a negative control. Osteogenic differentiation was analyzed after 14 days of cultivation using Alizarin Red S staining (Sigma Aldrich, Taufkirchen, Germany) according to the standard procedure. For chondrogenic differentiation, micromass cultures were generated by seeding 5 μL droplets containing 4 × 10^4^ cells into the middle wells of a 12-well plate. After the micromass cultures were cultured for 6 h under high humidity, chondrogenic medium (StemPro Chondrogenesis Differentiation Kit, Gibco, Grand Island, NY, USA) was added to the culture vessels. The cell culture medium was added to the wells, which served as a negative control. The micromass cultures were incubated at 37 °C in an incubator with 5% CO_2_ and a humid atmosphere. The medium was changed every 2–3 days. Chondrogenic differentiation was analyzed after 14 days of cultivation using Alcian blue staining (Sigma Aldrich, Taufkirchen, Germany) according to a standard procedure. The differentiated cells were visualized under a light microscope.

### 2.5 Biomaterials and media preparation

Three materials were tested on polystyrene-grown cDPSCs: (1) ProRoot^®^ MTA (Dentsply Sirona, Johnson City, TN, USA) (MTA), a calcium-silicate endodontic cement commonly used for pulp capping and root-end filling; (2) RS + ™ (GenTech – Genuine Technologies d.o.o., a spin-out of Jožef Stefan Institute, Ljubljana, Slovenia) (RS+), a synthetic bioceramic, calcium trisilicate-based powder with small additions of biocompatible phyllosilicate clay (bentonite) and bioactive amorphous calcium silicate for an enhanced handling, setting, and remineralization response, indicated for root canal repair and sealing; and (3) CellFoam™ (BioChange Ltd., Yokneam, Israel) (CF), a commercially available porous, cell-culture-grade biodegradable scaffold.

We prepared two different media, containing experimental materials, as exposure models:

- Suspension medium (to simulate acute cytotoxic conditions)

Each biomaterial was suspended in cell culture medium at an initial concentration of 50 mg/mL (defined as the first dilution, D1). Seven additional twofold serial dilutions were prepared (D1–D8). This powder-in-medium setup simulated the immediate, high-exposure environment that may occur after material placement, which is particularly relevant for calcium silicate-based cements (MTA and RS+), which can transiently release Ca(OH)₂, increase the pH, and directly contact surrounding cells with particulate matter.

- Conditioned medium (to simulate physiologically relevant conditions)

Each biomaterial was first suspended in culture medium at 50 mg/mL, shaken overnight at room temperature, and centrifuged the following day at 500 × *g* for 10 min. After the supernatant was collected, we adjusted its pH to 7.5 to isolate material-specific effects from pH-mediated cytotoxicity. To adjust the pH, we used 1 N HCl, as the buffering components of the culture medium were insufficient to counteract the high alkalinity resulting from Ca(OH)₂ release. The supernatant was then filtered through 0.22 μm syringe filters. The resulting conditioned medium was used for experimental cell culture at four twofold serial dilutions (D1–D4).

### 2.6 MTT assay (metabolic activity)

An MTT assay was employed for the suspension and conditioned media cultures. 3-(4,5-dimethylthiazol-2-yl)-2,5-diphenyltetrazolium bromide was used to measure cellular metabolic activity as an indicator of the cytotoxicity of the biomaterials. It is based on the reduction of a yellow tetrazolium salt (MTT) to purple formazan crystals by metabolically active cells. Cells frozen at passage 1 were thawed, seeded at passage 2, multiplied and reseeded at passage 3 for the MTT assay. Cells were seeded in quadruplicate into clear 96-well microtiter plates at a cell density of 10^4^ cells/cm^2^ in a final volume of 100 μL of culture medium. Cells were cultured at 37 °C in a 5% CO_2_ incubator for 48 h until they reached 70% confluency. After the incubation period, the cell culture medium was removed, and experimental medium was added. The cells were cultured overnight. The experimental medium was removed, and 10 μL of MTT labeling reagent (at a final concentration of 0.5 mg/mL) was added to 100 μL of DMEM without phenol red (Gibco, Grand Island, NY, USA) in each well. Following 4 h of incubation at 37 °C, in a 5% CO_2_ incubator, 100 μL of solubilization buffer was added to each well and incubated overnight at 37 °C in a 5% CO_2_ incubator. The next day, the total solubilization of the purple formazan crystals was measured with a Byonoy absorbance reader (Byonoy, Hamburg, Germany). The sample wavelength was set at 562 nm, and the reference wavelength was 650 nm.

### 2.7 Live/dead assay (viability)

A live/dead assay was employed for suspension and conditioned media cultures. Cells frozen at passage 1 were thawed, seeded at passage 2, multiplied and reseeded at passage 3 for the live/dead assay. Cells were seeded at a density of 10,000 cells/cm^2^ into 8-well glass chamber slides (Merck, Darmstadt, Germany) and cultured for 48 h until they reached 70% confluence. After the incubation period, the cell culture medium was removed, and experimental medium was added. The cells were cultured overnight, after which the experimental medium was removed. A live/dead cell imaging kit (488/570) (Thermo Fisher Scientific, Waltham, MA, USA) was added to the cells, which were then incubated for 15 min. The cells were observed under a fluorescence microscope (Nikon Eclipse 80i, Nikon) equipped with a Nikon Digital Sight DS-U2 camera. Images were captured in the NIS-Elements D3.2 Live quality program at 400 × magnification and qualitatively analyzed. To calculate the viability from live and dead cell counts, the 𝑡𝑜𝑡𝑎𝑙 𝑐𝑜𝑢𝑛𝑡 𝑜𝑓 𝑙𝑖𝑣𝑒 𝑐𝑒𝑙𝑙𝑠 and 𝑡𝑜𝑡𝑎𝑙 𝑐𝑜𝑢𝑛𝑡 𝑜𝑓 𝑑𝑒𝑎𝑑 𝑐𝑒𝑙𝑙𝑠 were added to determine the total cell number. Viability was calculated using the following formula:


$$\begin{array}{l} V=\text{of viable cells}\;X\;((\text{total count of test sample})/\\ \;(\text{total count of control sample})) \end{array}$$

### 2.8 Alizarin Red S staining (osteogenic readout)

An Alizarin Red S (ARS) staining assay of cDPSCs differentiated into osteogenic lineages was performed for conditioned media cultures. Cells frozen at passage 1 were thawed, seeded at passage 2, multiplied and reseeded at passage 3 for ARS staining. Cells were first differentiated into osteogenic lineages. For osteogenic differentiation, 15 × 10^4^ cells/cm^2^ were seeded into 6-well plates. At 70% confluency, the cell culture medium was exchanged with experimental medium. After 24 h, the experimental medium was removed. Osteogenic medium (StemPro Osteogenesis Differentiation Kit, Gibco, Grand Island, NY, USA) was added, and the medium was changed every 2–3 days. Osteogenic differentiation was analyzed after 14 days of cultivation using Alizarin Red S staining (Sigma Aldrich, Taufkirchen, Germany) according to the standard procedure. The cells were observed under a fluorescence microscope at 400 × magnification and qualitatively analyzed. In each well, 3 images were taken and processed with the ImageJ program.

### 2.9 Image analysis

Images for the live/dead and ARS staining assays were analyzed with the ImageJ program. Images were captured in the NIS-Elements D3.2 Live quality program. Images were captured at 40 × magnification. For the live/dead assay, 3 images of live cells and 3 images of dead cells were randomly selected from each well and quantitatively analyzed by measuring the areas of green (live) and dead (red) cells in each well and processed with the ImageJ program. In the ImageJ program, images were converted to binary types and then segmented using the DynamicThreshold_1d.class plugin (33), which displayed (max + min)/2 images. The area of particles larger than 100 μm^2^ was measured in each field view, and the total area covered by cells was calculated. For ARS staining, 3 images were randomly selected from each well and quantitatively analyzed by measuring the area of red particles (mineral deposits) in each well, after which the samples were processed with the ImageJ program. In the ImageJ program, the images were processed with background adjustment, separated with Color Deconvolution2 (34), segmented using the DynamicThreshold_1d.class plugin, displayed as (max + min)/2 images, and the red area was measured. Particles larger than 10 μm^2^ were measured in each field view. The total area of red particles was calculated.

### 2.10 RNA isolation

RNA was isolated from experimental and control cDPSCs. Cells were detached from the wells with a cell scraper. The cell suspension was removed from the wells and centrifuged at 240 × *g* for 4 min. The supernatant was discarded, and the pellet was flash-frozen in liquid nitrogen. The cell pellet was then homogenized with a homogenizer (IKA T10 basic, Staufen, Germany) in 350 μL of RLT lysis buffer (Qiagen, Hilden, Germany). Total RNA extraction was carried out with an RNeasy Plus Mini Kit (Qiagen) according to the manufacturer’s protocol. The amount of extracted total RNA was measured using a UV spectrophotometer (Thermo Fisher Scientific, Waltham, MA, USA) at 260/280 nm.

### 2.11 Reverse transcription and real-time qPCR

Two-step reverse transcription quantitative polymerase chain reaction (RT–qPCR) for experimental cDPSCs at the third dilution and positive control cDPSCs was performed. First, 2 μg of total RNA from each sample was reverse transcribed into cDNA using a High-Capacity cDNA Reverse Transcription Kit with RNase Inhibitor (Thermo Fisher Scientific, Waltham, MA, USA) according to the manufacturer’s protocol. Negative reverse transcription controls were included in each PCR run. All reactions were conducted in a total volume of 20 μL. The conditions for reverse transcription were as suggested in the manufacturer’s protocol: 25 °C for 10 min, 37 °C for 120 min, and 85 °C for 5 min. In the second step, relative quantification was performed using TaqMan Universal PCR Master Mix with UNG (Thermo Fisher Scientific, Waltham, MA, USA) and the TaqMan gene expression assays RunX2 and ALPL. TBP was used as a reference gene (Thermo Fisher). All the qPCR amplifications were conducted in triplicate in a total volume of 20 μL. cDNA (20 ng) was used as a template. Amplification was carried out in 96-well plates with a Light Cycler 96 (Roche Life Science) using the following program: 50 °C for 2 min, 95 °C for 10 min, and 40 cycles at 95 °C for 15 s and 60 °C for 60 s.

### 2.12 Statistical analysis

Statistical analysis was performed for cells isolated from three dental pulp tissues and grown on standard polystyrene surfaces (Table 1). All the statistical analyses were performed with GraphPad Prism version 9.5.0 for Windows (GraphPad Software, San Diego, CA, USA, www.graphpad.com, accessed on 15 April 2024).

All the data were log-transformed to normalize the data and residuals. The normality and lognormality of the residuals were checked with the Kolmogorov–Smirnov test.

All the data were log-transformed (Y = log(Y)) and normalized to the control samples to account for differences in the control samples (formula: value/baseline). For the MTT, live/dead and ARS staining assays, 2-way ANOVA was performed to analyze the differences between the experimental cell cultures. For qPCR, conditioned media cultures from the third dilution (D3) were used. The efficiency-corrected double delta Ct method was employed to normalize the gene expression values (35). The expression levels of RUNX2, ALPL and MMP13 were compared to the expression levels of RUNX2, ALPL and MMP13 in positive control cDPSCs, and the results were analyzed by one-way ANOVA.

Statistical significance was defined as *p* < 0.05.

## 3 Results

### 3.1 Isolation and characterization of cDPSCs

**Figure 1:**
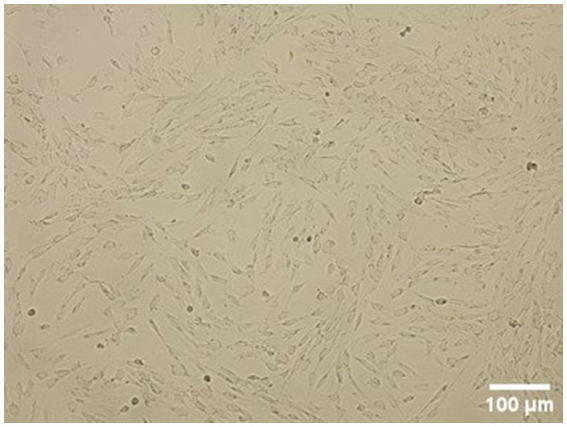
Morphology of cells grown on a standard plastic surface. cDPSCs from passage 3 are spindle shaped with a typical fibroblast-like morphology.

Dental pulp tissue was successfully collected from all three teeth of the two dogs. Under a light microscope, the cells from passage 3 appeared spindle-shaped with a fibroblast-like morphology (Figure 1).

### 3.2 Flow cytometry for surface marker expression

**Figure 2:**
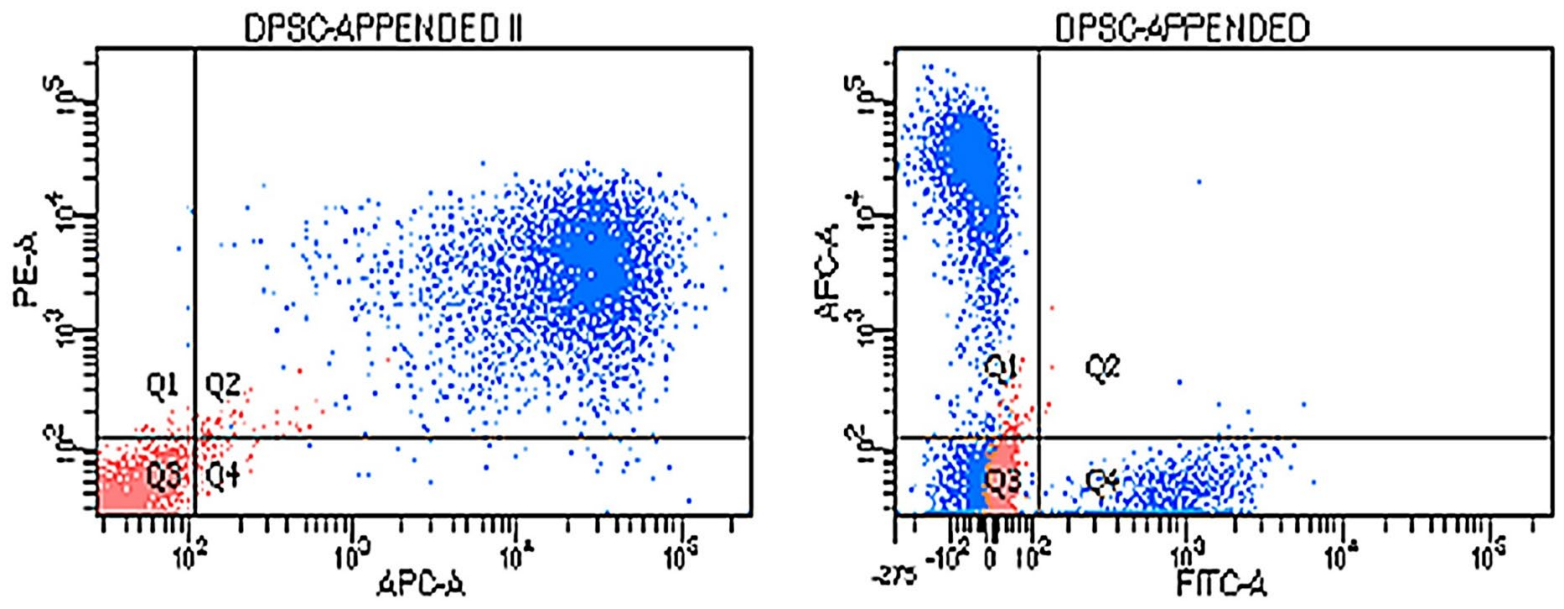
Expression of cDPSC surface markers. Blue cells are labeled with antibodies. Red cells are unlabeled cells. On the left dot plot are cells stained with the positive cell surface markers CD44-APC and CD90-PE (quadrant Q2). Unlabeled cells (red) are appended to quadrant Q3. On the right dot plot are cells positive for the cell surface marker CD44-APC (quadrant Q1) and CD29-FITC (quadrant Q4) and negative for the cell surface marker CD34-FITC (quadrant Q3). Unlabeled cells (red) are appended to quadrant Q3.

Flow cytometry was performed at passage 3 with conjugated primary antibodies against the positive surface markers CD44-APC, CD90-PE, and CD29-FITC and the negative surface marker CD34-FITC. The cells were positive for CD44, CD90, and CD29 but negative for CD34 (Figure 2).

### 3.3 Multilineage differentiation potential

**Figure 3:**
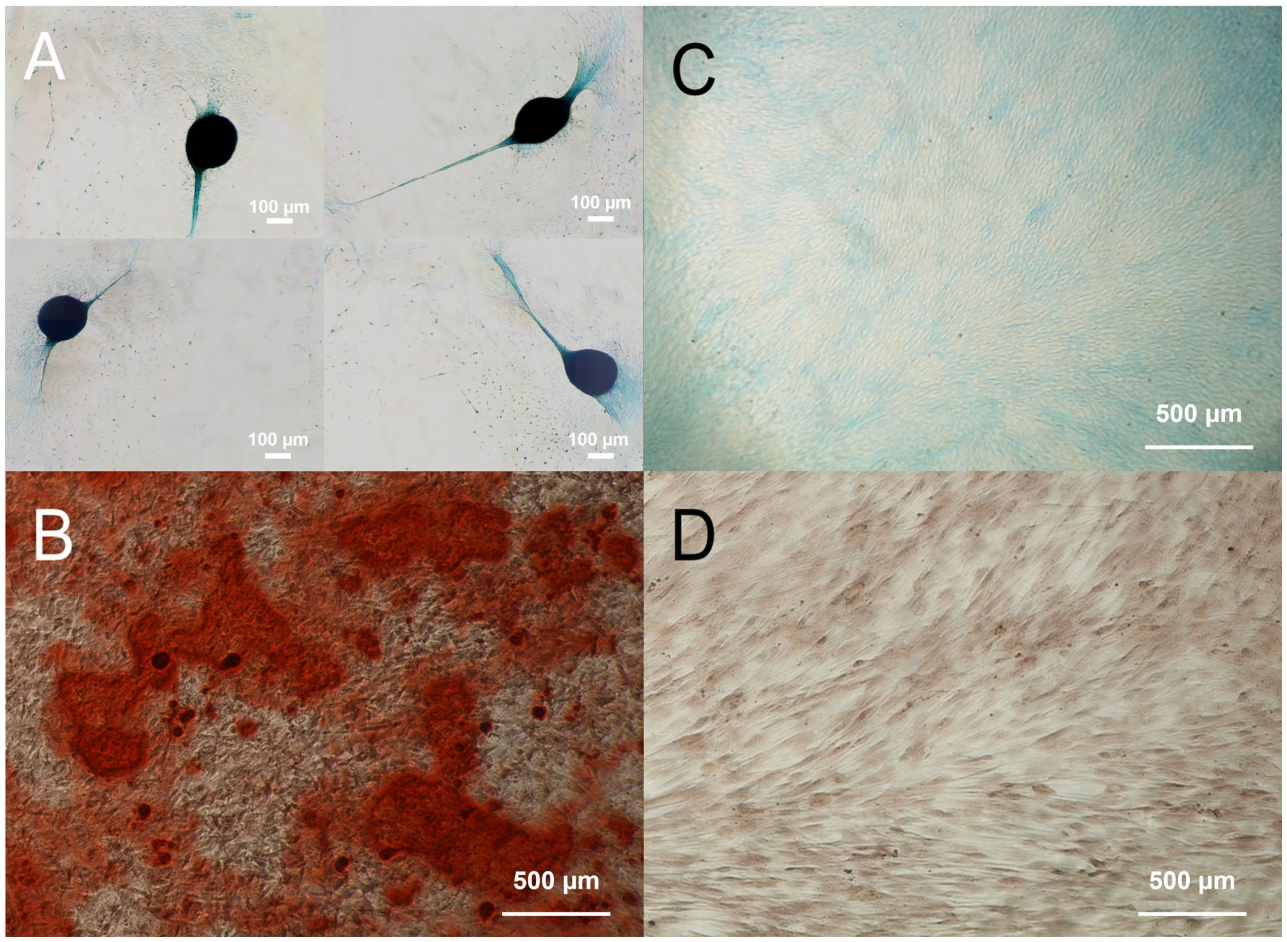
Chondrogenic (**A**; Alcian blue) and osteogenic (**B**; Alizarin Red S) differentiation of cDPSCs with corresponding negative controls **(C,D)**.

cDPSCs successfully differentiated into chondrogenic and osteogenic lineages. Chondrogenesis was indicated by the formation of chondrogenic nodules, which were stained blue with Alcian blue (Figure 3A). Mineral deposits in the extracellular matrix stained red with Alizarin Red S indicate osteogenesis (Figure 3B). The corresponding negative controls are shown on the right (Figures 3C,D).

### 3.4 Metabolic activity (MTT)

**Figure 4:**
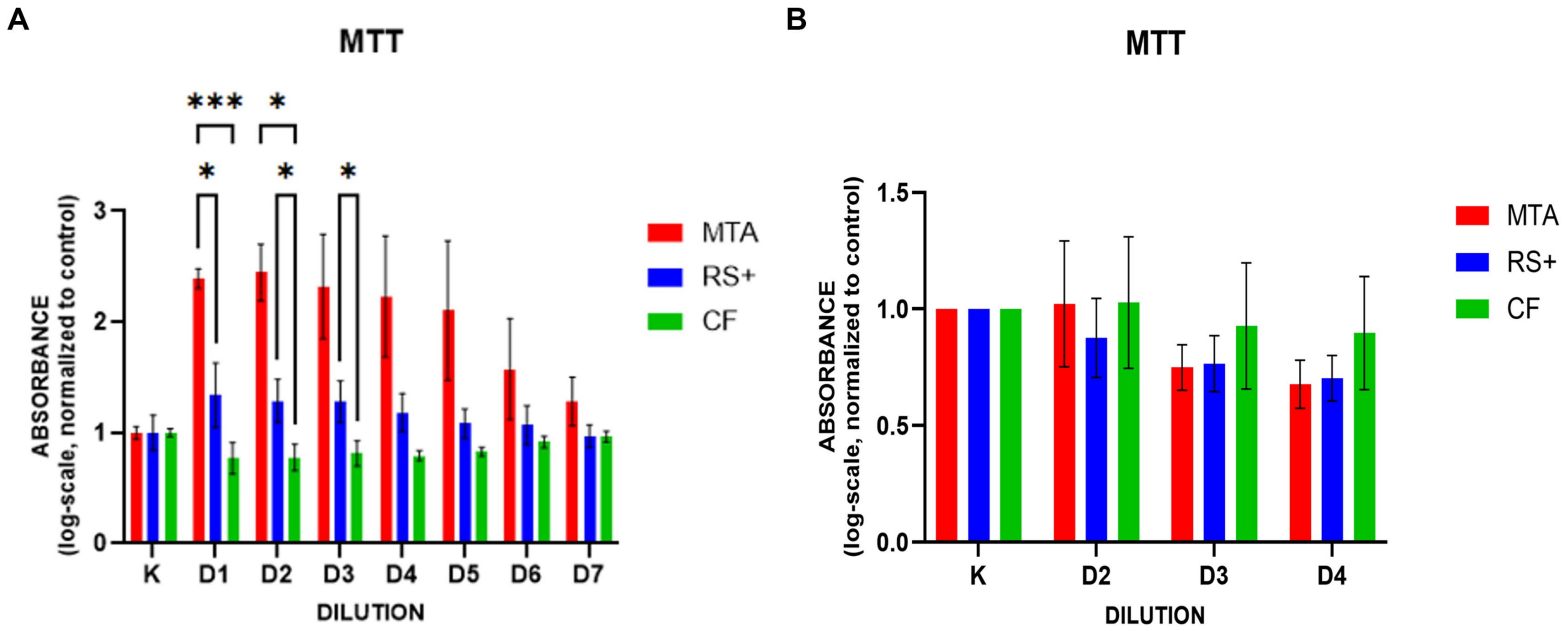
MTT assays in experimental media. In suspension culture **(A)**, log transformation of the data revealed that higher plotted values corresponded to lower absorbance (lower mitochondrial activity). In suspension culture, the MTT absorbance was greater with RS + than with MTA at D1 and greater with CF than with MTA at D1 and D2. CF also yielded higher values than RS + did at D2 and D3 (* *p* < 01, *** *p* < 0.001). In the conditioned media **(B)**, no significant differences in MTT absorbance were observed among the groups.

In suspension culture, the MTT absorbance (a proxy for viable cell number) was greater with RS + than with MTA at D1 and greater with CF than with MTA at D1 and D2. CF also yielded higher values than RS + did at D2 and D3 (Figure 4A). No significant differences in MTT absorbance were observed among the groups treated with conditioned media (Figure 4B). In the MTT with the conditioned media, dilutions 2, 3, and 4 were used; the first dilution was omitted to avoid introducing possible artifacts into the analysis because preliminary attempts using this dilution produced inconsistent absorbance values.

### 3.5 Viability (live/dead)

**Figure 5:**
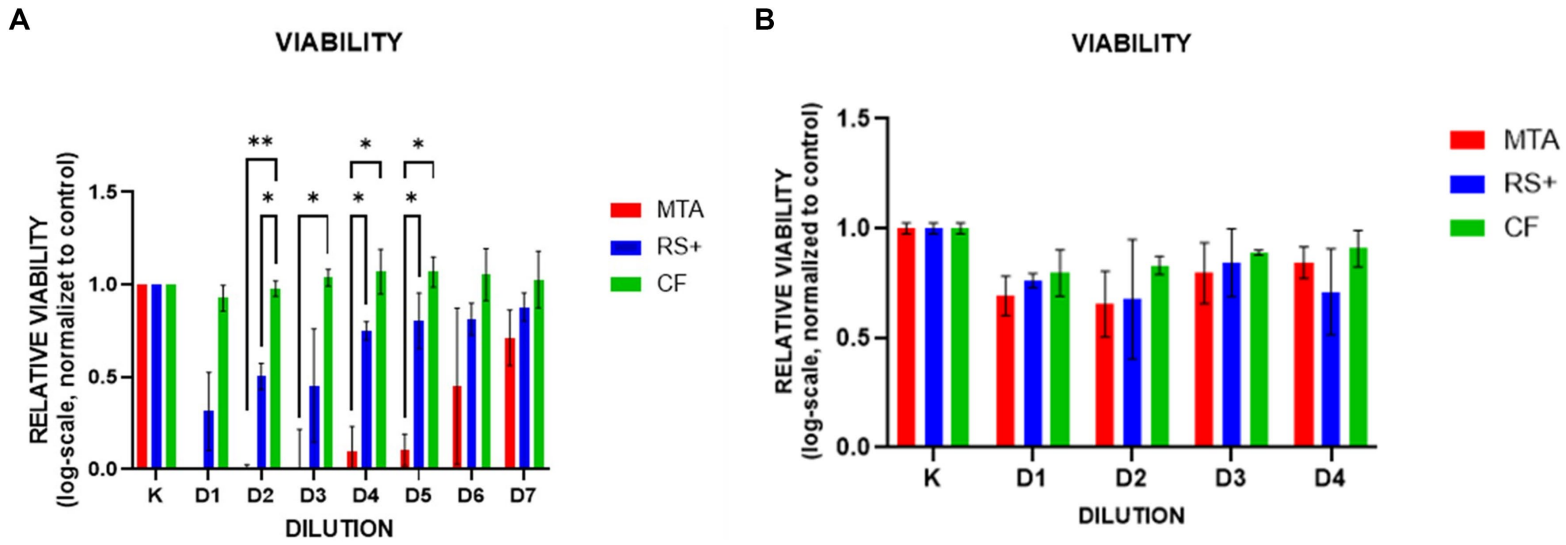
Viability in suspension culture across dilutions. In suspension media culture **(A)**, cell viability was greater with CF than with MTA at dilutions D2–D5 and greater with CF than with RS + at D2; RS + also exceeded MTA at D4 and D5. Exact significance levels are indicated on the plot (* *p* < 0.05; ** *p* < 0.01). In conditioned media culture **(B)**, no statistically significant differences in viability were observed among the groups.

In suspension culture, cell viability was greater with CF than with MTA at dilutions D2–D5 and greater with CF than with RS + at D2; RS + also exceeded MTA at D4 and D5 (Figure 5A). In the conditioned media, no statistically significant differences in viability were observed among the groups (Figure 5B).

### 3.6 Osteogenic outcome (Alizarin Red S)

**Figure 6:**
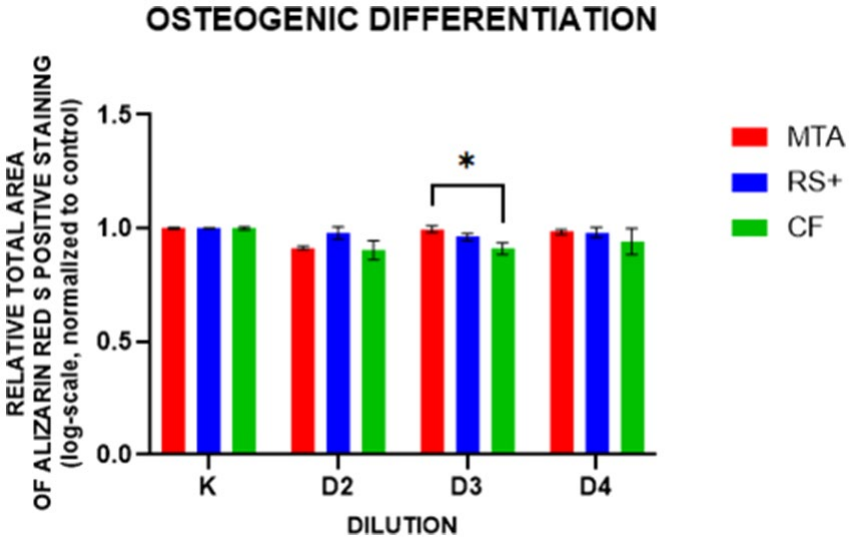
Relative mineralized areas after osteogenic induction of cDPSCs preexposed to conditioned media (D2–D4). Indicates *p* < 0.01 at D3 (MTA vs. CF).

**Figure 7:**
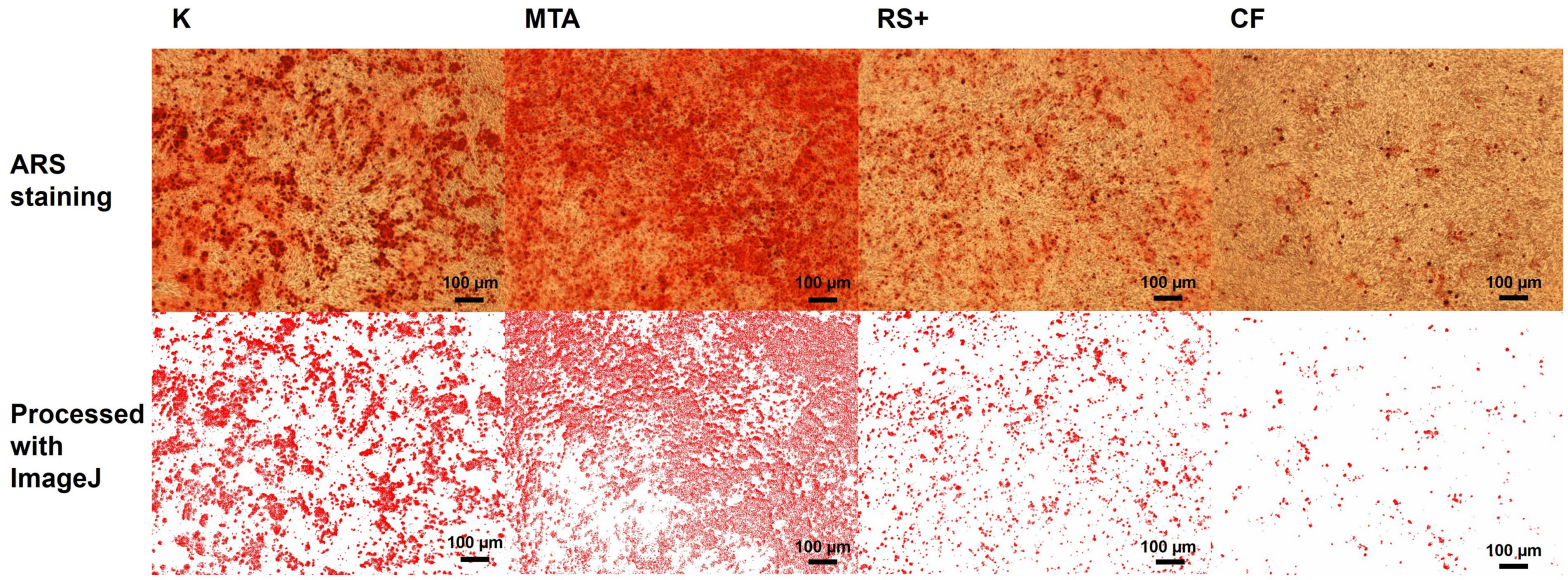
Representative images of ARS staining (upper row) and the corresponding images processed with ImageJ (lower row).

In the ARS, dilutions 2, 3, and 4 of conditioned media were used; the first dilution was omitted to avoid introducing possible artifacts into the analysis because preliminary attempts using this dilution produced uneven well-to-well staining. There was a difference in ARS staining at D3, where the ARS staining of cDPSCs was greater in cells conditioned with MTA than in cells conditioned with CF (Figure 6). Representative images of ARS staining and the corresponding images processed with ImageJ are shown in Figure 7.

### 3.7 Gene expression analysis (RT-qPCR)

**Figure 8:**
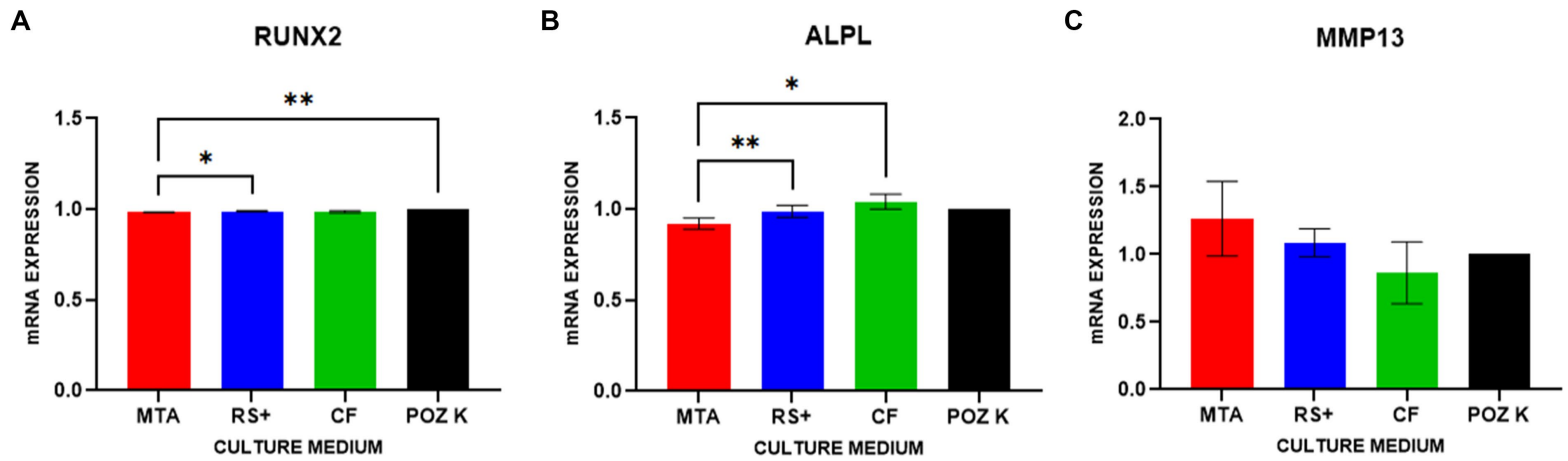
Gene expression in cDPSCs after conditioned media exposure followed by osteogenic induction. The expression of RUNX2 **(A)** was lower in cells conditioned with MTA than in cells conditioned with RS + (* *p* < 01) and lower than that in positive control cells (** *p* < 0.01). ALPL expression **(B)** was lower in cells conditioned with MTA than in cells conditioned with RS + (** *p* < 001) and CF (* *p* < 0.1). No differences in the expression of MMP13 **(C)** were observed between the groups.

As there was a significant difference in ARS staining on D3, gene expression was evaluated at this dilution. The expression of RUNX2 was lower in cells conditioned with MTA than in cells conditioned with RS + (* *p* < 0.1) and lower than that in positive control cells (** *p* < 0.01; Figure 8A). ALPL expression was lower in cells conditioned with MTA than in cells conditioned with RS + (** *p* < 0.01) and CF (* *p* < 0.1; Figure 8B). No differences in the expression of MMP13 were observed between the groups (Figure 8C).

## 4 Discussion

Stem cell-based regenerative therapies are being increasingly extended to dentistry and oral tissue regeneration. Dental pulp stem cells (DPSCs) are central to dentin–pulp repair and regeneration, and their beneficial effect has already been reported in a vital pulpectomy setting in dogs (36). In addition, DPSCs provide a relevant *in vitro* model for evaluating biomaterial interactions and their suitability for combined cell–material applications (29, 37).

To better understand how clinically established or emerging materials influence pulp-resident stem cells, we investigated the *in vitro* effects of three different materials on canine DPSCs (cDPSCs). The cDPSCs isolated in our study exhibited a typical mesenchymal phenotype (CD44^+^/CD90^+^/CD29^+^/CD34^−^) and multilineage potential, which is consistent with previous reports on cDPSCs (31, 32). We then compared the effects of ProRoot^®^ MTA (the clinical reference material in veterinary endodontics (38)), RS + ™ and CellFoam™ on the metabolic activity and cell viability of cDPSCs, as well as on their potential to undergo osteogenic and odontogenic differentiation. Two exposure paradigms were used to bracket the clinically relevant range of early material–tissue interactions. First, we modeled the initial, acute particle-associated cytotoxicity that may have occurred immediately after placement, simulating direct contact between freshly mixed material and adjacent pulp cells. Second, we assessed physiologically relevant, diffusion-controlled exposure using conditioned media (eluates), which better reflects the environment in which cells are present within tissue or when materials are combined with stem cell-laden scaffolds for regenerative applications. Assessing acute cytotoxicity is important because early interfacial chemistry (particularly Ca^2+^ and OH^−^ release and the resulting increase in pH) can strongly influence early cell survival, proliferation, and the onset of repair/regeneration. Materials that are excessively cytotoxic during this window could jeopardize pulp vitality or delay healing even if their long-term behavior is favorable once set (39). Although calcium-silicate cements such as MTA and RS + are typically applied freshly mixed with an appropriate diluent, such as deionized water or saline solution, and their interaction with pulp tissue begins immediately upon placement, unreacted particles with an ongoing topological transformation due to dissolution/recrystallization and subsequent ion release can transiently shift the microenvironment (40). Therefore, this study’s two-condition design aligns with prior *in vitro* work on bioceramic, hydraulic calcium-silicate cements, which often appear more cytotoxic when freshly mixed but become highly biocompatible after setting or when tested as eluates (41–43). Related animal and clinical studies similarly report that any transient irritation immediately after placement subsides as the material hydrates, with ultimate support for pulp healing, dentin bridge formation, and tissue integration (44–46).

We used an MTT assay to measure cellular metabolic activity as an indicator of the cytotoxicity of the biomaterials. In conditioned medium culture, compared with the cells grown in MTA medium, the cells grown in RS + and CF media showed higher mitochondrial activity in the early stages, particularly at D1, suggesting higher initial metabolic activity. The higher metabolic activity of cells grown in the CF medium than in the RS + medium at D2 and D3 further indicates that metabolic activity is more prolonged when the cells are cultured with CF. This pattern aligns with the early alkalinity and ion release of hydraulic calcium-silicate cements, which can transiently depress metabolism at higher effective concentrations (41–43). Under conditioned exposure, intermaterial differences diminished or disappeared, indicating that dilution, buffering, and partial setting modulate chemistry to levels compatible with those of pulp cells (44–46). From a clinical perspective, these dynamics are expected. *In vivo*, dentin and tissue fluids buffer the strong alkalinity of freshly mixed cement while the material hydrates and sets. Dentin’s hydroxyapatite (phosphate- and carbonate-substituted) mineral phases and organic matrix adsorb ions and favor calcium–phosphate precipitation, lowering effective hydroxyl and calcium ion activity at the interface (47–49). Tubular diffusion and pulpal fluid flow disperse ions further, and progressive hydration reduces reactivity over time (50–52). These mechanisms explain why freshly mixed MTA may appear to be cytotoxic *in vitro* but is well tolerated clinically.

The results of the live/dead assay were similar to those of the MTT analysis. Under acute suspension exposure, compared with MTA (dilutions D2–D5) and RS + (D2), CF consistently increased cell viability, whereas compared with MTA, RS + promoted greater cell viability at dilutions D4–D5. These findings confirm the low acute cytotoxicity of CF and its favorable interaction with cDPSCs. The reduced viability observed with suspended MTA likely reflects its transiently high alkalinity and rapid Ca(OH)₂ release, which can exceed physiological tolerance and compromise cell membrane integrity during early exposure (39, 52). In contrast, under conditioned medium exposure, the differences in viability among the materials were no longer significant, indicating that dilution and buffering during conditioning effectively mitigated the initial cytotoxic effects.

Taken together, the results of the MTT and live/dead assays support the concept that set or preconditioned calcium-silicate materials become highly biocompatible once the early reactive phase subsides, which is consistent with clinical observations of pulp healing following transient initial irritation (45, 46). Compared with MTA and RS + cells, cells cultured in CF maintained higher metabolic activity and viability in suspension cultures across multiple dilutions. These results indicate that CF has lower cytotoxicity and could therefore function as an immediate delivery vehicle for cDPSCs at the time of pulp capping or regenerative endodontic therapy. Specifically, in veterinary regenerative endodontics, 3D scaffolds (e.g., silk fibroin) are being increasingly explored as adjuncts that host cells, enable nutrient diffusion, and stabilize the microenvironment, whereas hydraulic cements provide the seal (53–55). In practice, such combinations may enhance tissue healing and bridge formation. The feasibility of stem cell-mediated pulp regeneration using cell-seeded scaffolds has been demonstrated in several *in vivo* studies in animal models. In a canine model, Bio-Oss scaffolds loaded with autologous DPSCs successfully supported the regeneration of periodontal and pulp-like tissues within experimental defects (56). Similarly, (76) reported that the delivery of DPSCs within scaffolds and their implantation into the root canals of dogs promoted the regeneration of vascularized pulp-like tissue. The formation of a dentin–pulp complex was also observed when collagen scaffolds were implanted with DPSCs, whereas cell-free scaffolds failed to induce such regeneration (57). Other cell–material combinations have also been explored in regenerative endodontics with promising results. The results of our study suggest that CF could also be a suitable carrier for cDPSC-based regenerative endodontic strategies.

Furthermore, we assessed the effect of clinically relevant conditioned medium exposure on cDPSC differentiation potential. To assess the mineralization ability of the cells, we differentiated cells from conditioned media culture into osteogenic lineages, as osteogenic and odontogenic lineages share overlapping molecular pathways (58, 59). Here, Alizarin Red S (ARS)-positive staining was used as a marker of early mineralizing (odontoblast-like) activity (60). All three tested materials in this study supported ARS-positive mineral deposition following osteogenic induction, with a difference observed only in dilution D3, where MTA exceeded CF. These findings agree with previous reports showing that when tested as extracts or after partial setting, MTA and similar materials yield comparable alkaline phosphatase activity and mineralization and tend to converge in performance once they are set or sufficiently diluted (61–66). ARS staining, together with increased expression levels of RUNX2, ALPL, and MMP13, supports a shift toward an odontoblast-like, mineralizing phenotype (67). Gene expression analysis was conducted only at D3 to examine how genes were regulated under the same conditions in which a difference in ARS staining was observed. By focusing on the dilution that resulted in a detectable change in mineralization, we aimed to determine whether transcriptional responses aligned with mineralization outcomes. Therefore, the gene expression findings refer specifically to this dilution. In contrast to the results of ARS staining, where CF performed comparably to calcium-silicate materials, we observed differences in the expression levels of genes in cDPSCs cultured in MTA-, RS + -, and CF-conditioned media following osteogenic differentiation. We tested three genes—RUNX2 (key regulator of osteogenic differentiation and early tooth development (68)), ALPL (commitment/mineralization), and MMP13 (a collagen-remodeling enzyme implicated in dentin matrix organization). RUNX2 is endogenously expressed in preodontoblasts, where it promotes lineage commitment; however, its expression must be downregulated for cells to progress toward terminal differentiation. This downregulation is essential for both the maturation of osteoblasts and the terminal differentiation of odontoblasts (69). Interestingly, in our study, the expression of RUNX2 was lower in cells conditioned with MTA than in cells conditioned with RS + and lower than that in positive control cells. The observed lower expression of RUNX2 in cells conditioned with MTA than in those conditioned with RS + and the positive control suggests that cells exposed to MTA-conditioned media may have already progressed beyond the preosteogenic/odontoblastic stage toward terminal differentiation and thus may reflect a more advanced stage of osteogenic/odontogenic differentiation of MTA-cultured cells rather than impaired lineage commitment. In contrast, the relatively high RUNX2 expression levels in the RS + and CF groups could indicate that these conditions maintained the cells in an earlier differentiated state. Like that of RUNX2, the expression of ALPL was lower in cells conditioned with MTA than in cells conditioned with RS + and CF. ALPL is a mineralization-associated marker gene (70, 71) and regulates the odontoblastic differentiation of DPSCs (37). Moreover, it is an early marker of osteogenesis, and its activity decreases as mineralization occurs (72). The observed higher expression levels of ALPL in the RS^+^ and CF groups therefore might reflect differences in temporal progression, with cells in the MTA group already entering an active mineralization phase, whereas those in the RS^+^ and CF groups remained in earlier or transitional stages of differentiation. This interpretation also aligns with the RUNX2 expression pattern. No differences in MMP13 expression were observed between the groups. MMP13 plays important roles in tooth development, odontogenic differentiation, and dentin–pulp reparative mechanisms (73). MMP13 is involved in tertiary reactionary dentin formation after tooth injury *in vivo*, potentially acting as a key molecule in the dental pulp during dentin–pulp repair processes and organizing and regulating dentin–pulp reparative processes (74). In our study, we detected no differences in MMP13 expression between the groups, suggesting that all three tested materials supported comparable levels of matrix remodeling activity or that MMP13 regulation was not strongly influenced by the moderate chemical differences among the conditioned media.

Taken together, the differentiation potential results indicate that all three tested materials—MTA, RS + and CF—support the osteogenic/odontogenic differentiation of cDPSCs under clinically relevant, conditioned conditions. ARS staining confirmed comparable mineral deposition across materials, and gene expression analysis of RUNX2 and ALPL suggested that cells cultured in MTA-conditioned medium may have progressed to a more advanced stage of differentiation than those exposed to RS + or CF. These results are in line with those of veterinary studies reporting high vital pulp therapy success, with consistent hard-tissue bridge formation and maintained pulp vitality in dogs (44–46), supporting the translational relevance of our *in vitro* results.

A limitation of this study is the small donor sample size, which may have contributed to biological variability and could limit the generalizability of the results. Additionally, age and tooth developmental stage can affect dental pulp regeneration (75). Therefore, larger, age-balanced donor cohorts will help refine effect sizes and reduce variability in future studies. Most importantly, future *in vivo* studies in clinically relevant models are crucial to translate the *in vitro* findings into clinically applicable outcomes (Table 3).

**Table 3:** QPCR assays and justification for gene selection in odontogenesis.

Gene symbol	Gene name	Assay ID (*Canis familiaris*)
RUNX2	Runt-related transcription factor 2	Cf02694692_m1
ALPL	Alkaline phosphatase	Cf02732788_uH
MMP13	Matrix metalloproteinase-13	Cf02741638_m1
TBP	TATA-box binding protein	Cf02637231_m1

## 5 Conclusion

In summary, this study demonstrated that MTA, RS + and CF are biocompatible with cDPSCs and support their metabolic activity, viability and differentiation under clinically relevant exposure conditions. All three materials supported comparable mineralization with gene expression patterns, suggesting a more advanced differentiation stage in MTA-conditioned cells, which is consistent with the findings of current veterinary studies reporting high success rates of vital pulp therapy with MTA. In contrast, in acute exposure culture media, compared with calcium-silicate materials, CF maintained higher cell viability and metabolic activity, indicating its potential as a carrier for DPSC delivery in stem cell-based regenerative endodontic strategies.

## Data Availability

The original contributions presented in the study are included in the article/supplementary material, further inquiries can be directed to the corresponding author.
